# Improved Assay for Quantifying a Redox Form of Angiotensinogen as a Biomarker for Pre-Eclampsia: A Case-Control Study

**DOI:** 10.1371/journal.pone.0135905

**Published:** 2015-08-27

**Authors:** Soheila Rahgozar, Tayebeh Amirian, Miao Qi, Zahra Shahshahan, Mansureh Entezar-E-Ghaem, Hatav Ghasemi Tehrani, Mehran Miroliaei, Steven A. Krilis, Bill Giannakopoulos

**Affiliations:** 1 Department of Biology, Faculty of Science, University of Isfahan, Isfahan, Iran; 2 Department of Infectious Diseases, Immunology and Sexual Health and Department of Medicine, St George Hospital, University of New South Wales, Sydney, Australia; 3 Department of Obstetrics and Gynaecology, Shahid Beheshti Hospital, Isfahan University of Medical Sciences, Isfahan, Iran; Indiana University School of Medicine, UNITED STATES

## Abstract

**Objective:**

Angiotensinogen exists in two distinct redox forms in plasma, the oxidized sulfhydryl-bridge form and the reduced, unbridged, free thiol form. The oxidized form of angiotensinogen compared to the free thiol form preferentially interacts with renin resulting in increased generation of angiotensin. The predictive potential of the ratio of free-thiol to oxidized angiotensinogen in the plasma for pre-eclampsia was first suggested by the Read group in ref 10. We propose an improved method for determining the ratio and validate the method in a larger cohort of pregnant women.

**Methods:**

Plasma samples from 115 individuals with pre-eclampsia and from 55 healthy pregnant control subjects were collected sequentially over a 2 year period. Using two distinct enzyme-linked immunosorbent assays (ELISAs) the plasma levels of total and free thiol angiotensinogen were quantified. The oxidized angiotensinogen plasma level is derived by subtracting the level of free thiol, reduced angiotensinogen from the total angiotensinogen levels in the plasma.

**Results:**

The relative proportion of free thiol angiotensinogen, expressed as a percentage of that observed with an in-house standard, is significantly decreased in pre-eclamptic patients (70.85% ± 29.49%) (mean ± SD) as compared to healthy pregnant controls (92.98 ± 24.93%) (mean ± SD) *p* ≤ 0.0001. The levels of total angiotensinogen did not differ between the two groups.

**Conclusion:**

Patients with pre-eclampsia had substantially lower levels of free thiol angiotensinogen compared to healthy pregnant controls, whilst maintaining similar total angiotensinogen levels in the plasma. Hence, elevated levels of plasma oxidized angiotensinogen may be a contributing factor to hypertension in the setting of pre-eclampsia.

## Introduction

Pre-eclampsia is a multisystem disorder of pregnancy characterized by hypertension and proteinuria that usually manifests after 20 weeks gestation [1]. It can affect 2–5% of pregnancies in developed countries [1]. It is more common in primigravid pregnancy, and is associated with significant maternal and fetal morbidity and mortality. Some patients with pre-eclampsia may develop liver dysfunction, thrombocytopenia, and hemolysis and are classified as having HELLP syndrome [2]. An important aspect of treating pre-eclampsia entails timely delivery of the baby and removal of the placenta [1,3], which suggests a pivotal role of the placenta in contributing to the disease process.

A precise understanding of pre-eclampsia pathogenesis remains elusive. Dysregulated invasion of endometrial arteries by cytotrophoblasts may contribute to disrupted placental perfusion by the spiral arteries [4,5]. It has been postulated that the spiral artery abnormalities encountered in the placenta of pre-eclamptic patients may predispose to hypoxia-reperfusion injury in the placental milieu, and the concomitant generation of an excess load of free radicals and reactive oxygen species (ROS) in the maternal systemic circulation [6]. The free sulfhydryl group of aminothiols such as cysteine, homocysteine, cysteinylglycine and glutathione constitute an important component of the body’s anti-oxidant defense system by scavenging free radicals, which results in the formation of disulfides (oxidized thiols) [7]. The free thiol-to-oxidized ratios for cysteine, cysteinylglycine and homocysteine have been found to be lower in women with pre-eclampsia compared to healthy pregnant controls [7].

The renin-angiotensin system plays an important role in salt and water homeostasis and in the maintenance of vascular tone [8]. Renin cleaves the amino-terminus of angiotensinogen to generate angiotensin I, which, after cleavage by the angiotensin-converting enzyme (ACE), is converted to angiotensin II, a peptide hormone that promotes vasoconstriction and sodium retention [9]. Medications based on inhibition of the renin-angiotensin system are an important treatment modality for hypertension.

Angiotensinogen exists in a 2:3 ratio of free thiol-to-oxidized angiotensinogen in the plasma of healthy individuals, independent of age and gender [10]. The conformational change from the free thiol to the oxidized form facilitates an increase in the generation of angiotensin I [10]. Of note, using a Western blotting method, elevation of oxidized angiotensinogen plasma levels relative to free thiol angiotensinogen levels have been found in 12 pre-eclamptic patients compared to 12 healthy pregnant control women (10). Western blotting however cannot quantify the levels of the two redox forms of angiotensinogen and at best is semi quantitative [10].

**Fig 1:**
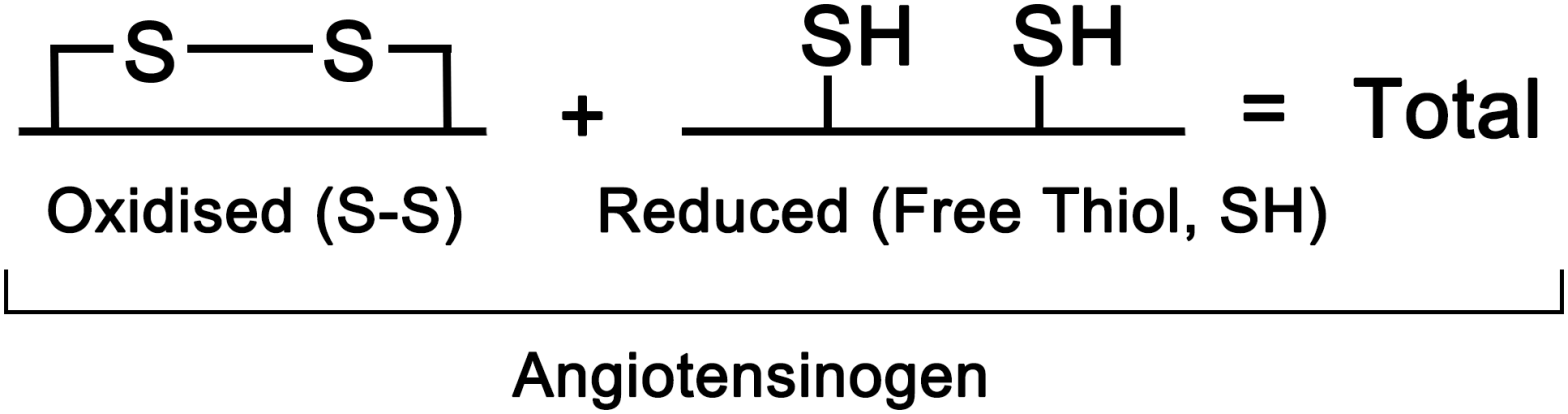
Total angiotensinogen plasma level is the sum of the plasma levels of the oxidized and reduced forms of angiotensinogen.

The current study evaluates novel and relatively simple ELISAs for quantitating free thiol and total angiotensinogen levels in a large case-control study. Decreased plasma levels of free thiol angiotensinogen, and as a corollary a corresponding increase in plasma oxidized angiotensinogen levels were found in pre-eclamptic patients compared to healthy pregnant controls. Total angiotensinogen levels (the sum of free thiol angiotensinogen and oxidized angiotensinogen plasma levels) (Fig 1) did not differ between the pre-eclamptic and healthy pregnant women.

## Materials and Methods

Hank’s Balanced Salt Solution (HBSS), HEPES, Tween 20, phosphate buffered saline (PBS) tablets, acetone for HPLC > 99.9% and p-nitrophenyl phosphate disodium salt hexahydrate (PnPP) tablets were purchased from Sigma-Aldrich, St. Louis, MO. *N*-(3-maleimidylpropionyl) biocytin (MPB) was from Invitrogen, Carlsbad, CA and Immobilizer Streptavidin C8 and MaxiSorp 96-well plates were purchased from Nunc, Roskilde, Denmark. Polyclonal rabbit anti-human angiotensinogen antibody (pAb) (catalogue number LS-C80597) was purchased from Lifespan Bioscience (Seattle, WA). Polyclonal goat anti-mouse IgG (Fab specific) alkaline phosphatase (AP) antibody (catalogue number A1293), polyclonal goat anti-rabbit IgG (whole molecule) AP (catalogue number A9919), bovine serum albumin (BSA) and angiotensinogen from human plasma were purchased from Sigma-Aldrich. Mouse anti-human angiotensinogen monoclonal antibody (clone 369439) was purchased from R&D Systems, Minneapolis, MN.

### Sample collection

Blood samples were collected sequentially at the time of enrolment, over a 2 year period, through Shahid Beheshti Hospital (Isfahan, Iran) in collaboration with the University of Isfahan. 3mls of blood was collected from each person with full written informed consent and in compliance with the ethical protocol and standards of Shahid Beheshti Hospital. In order to separate plasma, blood samples were collected into tubes with EDTA and centrifuged (917*g*) at 4°C for 10 min. The prepared plasma samples were stored at −20°C until use.

### Enrolment of Study Subjects

Plasma samples from 115 individuals with pre-eclampsia and 55 healthy pregnant control subjects, were collected sequentially, at the time of enrolment.

The sample size was calculated according to the following formula:

$$\text{n}={\left({\text{Z}}_{1-\text{α}/2}+{\text{Z}}_{1-\text{β}}\right)}^{2}\left(\text{S}1^{2}+\text{S}2^{2}\right)/{\text{d}}^{2}$$

n = sample size

α and β = 0.01 and 0.05 for 99% and 95% confidence intervals, respectively

S1 and S2 = 0.088 and 0.154, standard deviations for free thiol angiotensinogen levels of patients and control samples, respectively. Numbers are extracted from the pilot study performed on 14 samples (7 patients and 7 controls) by our group.

d = 0.1, margin of error

The inclusion criteria for diagnosing pre-eclampsia were based on the American College of Obstetricians and Gynecologists (ACOG) clinical guidelines, 2013 [11]: a) high blood pressure (≥ 140/90 mm/Hg) on at least 2 occasions ≥ 4 h apart, but not >7 days apart and either b) significant proteinuria (excretion of ≥ 300 mg protein in a 24 h urine collection) after 20 weeks of pregnancy or c) in the absence of proteinuria, the new onset of any of the following: thrombocytopenia, renal insufficiency, impaired liver function, pulmonary edema, or cerebral or visual symptoms. All the patients included in our study had hypertension and proteinuria but not the features listed in c). The healthy subjects had no history of fetal death (i.e., death of conceptus ≥ 10 weeks gestation), no more than 1 miscarriage less than 10 weeks gestation, and no history of positive antiphospholipid antibodies. Cases and controls were non-smokers.

### ELISA to detect total angiotensinogen

In order to detect the total angiotensinogen levels in the plasma samples, an in house ELISA was developed. MaxiSorp 96-well plates were coated with 50 μl commercial mouse-anti-human angiotensinogen mAb (10 nM/well) in 50 mM carbonate–bicarbonate buffer, pH 9.6 and incubated at 4°C for 14 h. After washing (4 times with PBS 0.1% Tween-20) and blocking the wells with 2% BSA/PBS 0.1% Tween-20, 50 μl/well of diluted plasma (1:1 in PBS 0.05% Tween-20) was added in duplicate and incubated at room temperature (RT) for 1 h. The second Ab, rabbit-anti-human angiotensinogen pAb (20 nM/well, diluted in 0.25%BSA/ PBS 0.1% Tween-20) was added after washing the plate and incubating at RT for 1 h. Following further washing of the plates, 50 μl of AP-conjugated goat-anti-rabbit IgG (1:1,500/well, diluted in 0.25% BSA/PBS 0.1% Tween-20) was added and incubated at RT for 1 h. After washing 3 times, chromogenic substrate was added and the generation of p-nitroaniline was monitored by measuring the OD at 405 nm. The concentration of plasma angiotensinogen was derived from a standard curve constructed with known concentrations of purified angiotensinogen (0.7–70 μg/ml). Linearity within this assay was achieved between dilutions 0 and 100 μg /ml of a pooled plasma sample composed of 20 plasma non-pregnant control samples.

Intra-plate coefficient ofvariation (CV) for this ELISA was calculated by running duplicates of 15 patient and control samples on a single plate. Inter-plate CV was calculated by running pooled plasma (1:1) in duplicate in every ELISA experiment.

### ELISA to detect free thiol angiotensinogen

**Fig 2:**
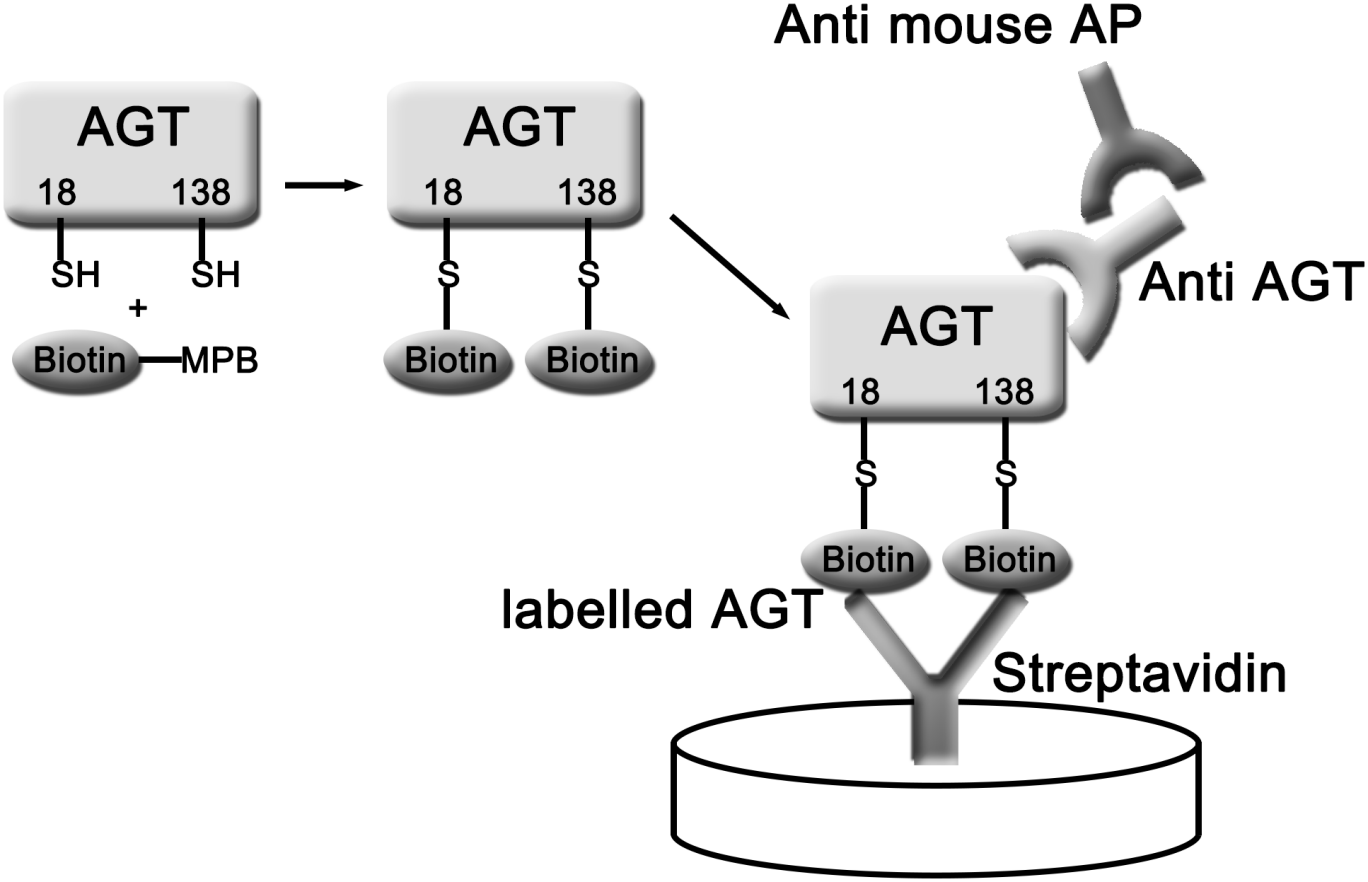
ELISA method for measuring free thiol angiotensinogen plasma levels. Biotin-MPB is added to plasma which labels free thiol (SH) angiotensinogen (ATG). The unincorporated Biotin-MPB is removed by acetone precipitation. Biotin-MPB labelled free thiols of ATG are specifically bound by streptavidin coated plates and bound free thiol ATG is quantified with a specific monoclonal antibody to ATG.

Measurement of the amount of free thiol angiotensinogen was based on labeling of free thiols in this protein with the biotin-conjugated selective free thiol binding reagent MPB, capturing biotin-labeled proteins on a streptavidin plate, and detecting the presence of MPB-labeled angiotensinogen with a specific anti-angiotensinogen mAb (Fig 2).

#### a) Labeling of plasma protein free thiols with biotinylated MPB

Modification of a previously published method to label free thiols in beta 2-glycoprotein I and factor XI was used [12–14]. Briefly, biotinylated thiol binding reagent MPB (1 μl) was added to 25 μl of individual plasma samples under argon and incubated at RT in the dark with agitation for 30 min. 16 μl from each MPB labeled sample was then diluted in 784 μl HBSS and further incubated at RT for 5 min. Acetone precipitation was used to remove unbound MPB from plasma samples. 1.6 ml ice-cold acetone was added to 400 μl of diluted sample. Samples were then incubated over night at -20°C. They were then centrifuged twice at high speed (5130 *g*) at 4°C for 25 min and the pellets were resuspended into 1.6 ml PBS 0.05%Tween-20.

#### b) Quantification of MPB labeled angiotensinogen by ELISA

MPB labeled samples were processed as previously described [15], briefly, they were added in duplicate to a streptavidin-coated 96-well plate that had been blocked with 2% BSA/PBS–0.1% Tween-20 (100 μl/well) and incubated at RT for 90 min. After washing 4 times with PBS 0.1% Tween-20, the rabbit anti-human angiotensinogen was added (100 μl /well, 1:500) and incubated at RT for 1 h. After washing 4 times with 230 μl PBS 0.1% Tween-20, AP conjugated anti-goat IgG was added (1:1500) and incubated at RT for 1 h. After washing 4 times with PBS 0.1% Tween-20 and addition of the chromogenic substrate pNPP (100 μl/well), the plate was covered with foil and incubated in the dark for 55 min at 37°C. The optical density of samples was read at 405 nm. Intra-plate CV was calculated by running duplicates of 8 samples on a single plate and calculating degree of MPB labeling for each well as a percentage of that observed with quadruplicate of the pooled plasma sample (on the same plate). For calculation of inter-plate CV, the same ELISA was performed on four different plates by running quadruplicates of the same sample, on different days. The individual performing the ELISA assays was blinded to the clinical status of the pregnant women.

There is no commercially available free thiol angiotensinogen standard that allows for the construction of a linear curve using different concentrations of purified free thiol angiotensinogen. So instead free thiol angiotensinogen results are expressed as a percentage of normal pooled plasma which constitutes an internal control and standard. Briefly, each patient or control plasma sample is labeled with a biotin specific probe which labels free thiols. To control for variation in labeling of plasma samples normal pooled plasma control is labeled at the same time and assayed on every ELISA plate.

### Statistical Analyses

Analyses were performed on GraphPad Prism 5 and SPSS 16 software (San Diego, CA). Differences were assessed through independent t-test (two-tailed). *p*<0.05 is considered significant. Area under the curve (AUC) statistics and 95% confidence intervals were recorded for the ROC curve analyses. Cutoff point for each model was defined as the coordinate that had the closest value to 1 for the difference between the true positive (sensitivity) and false-positive (1-specificity) values.

## Results

### A) Demographic, clinical details and laboratory data of the patients with pre-eclampsia and the healthy pregnant control group are summarized in Tables 1 and 2

**Table 1:** Demographic characteristics of the patient and control groups.

	Patient	Control	P value
**Number**	**115**	**55**	
**Age(y)**	**28.4±5.1**	**26.6±4.6**	**0.06**
**Gestation age(w)**	**38.5±3.2**	**39.4±1.05**	**0.76**
**Weight(Kg)**	**77.1±9.7**	**83.8±5.2**	**0.35**
**Stature(cm)**	**159.8±10**	**161±5.4**	**0.25**
**BMI**	**30.2±8.3**	**32.3±4.0**	**0.19**
**Parity status**	**1.50±0.65**	**1.38±0.58**	**0.51**

BMI = body mass index, Parity status = number of pregnancies including the current pregnancy

**Table 2:** Clinical and laboratory characteristics of the patient and control groups.

Clinical and Laboratory Parameters	Patient	Control	P value
**Number**	**115**	**55**	
**Systolic BP(mmHg)**	**149.2±8.4**	**112±5**	**0.0001**
**Diastolic BP(mmHg)**	**96±5**	**75.5±6**	**0.0001**
**LDH(U/L)**	**460±132.9**	**375±92.5**	**0.43**
**SGOT(U/L)**	**24±11.6**	**18.5±4.2**	**0.42**
**SGPT(U/L)**	**19± 13.8**	**12±6.8**	**0.19**
**Bilirubin total(mg/dl)**	**0.41±0.3**	**0.4±0.13**	**0.37**
**BUN (mg/dl)**	**9.4±3.3**	**9.1±2.9**	**0.06**
**Creatinine(mg/dl)**	**1.03±0.2**	**0.94±0.1**	**0.25**
**Hemoglobin(g/dl)**	**13.5±1.6**	**12±1.7**	**0.06**
**Hematocrit(%)**	**38.1±11.4**	**37.8±4.4**	**0.73**
**Platelet count(x10** ^**3**^ **/μl)**	**198.5±82.9**	**206 ±55**	**0.89**

BP = Mean blood pressure on at least 2 occasions ≥ 4 h apart, but not >7 days apart; LDH = lactate dehydrogenase; SGOT = Serum glutamic oxaloacetic transaminase; SGPT *=* serum glutamic-pyruvic transaminase; BUN = blood urea nitrogen

Potential confounding factors including, age, gestation age, body mass index (BMI) and parity status were equivalent in the control and patient groups.

### B) Total angiotensinogen levels are not significantly different in healthy pregnant controls and pre-eclamptic women

**Fig 3:**
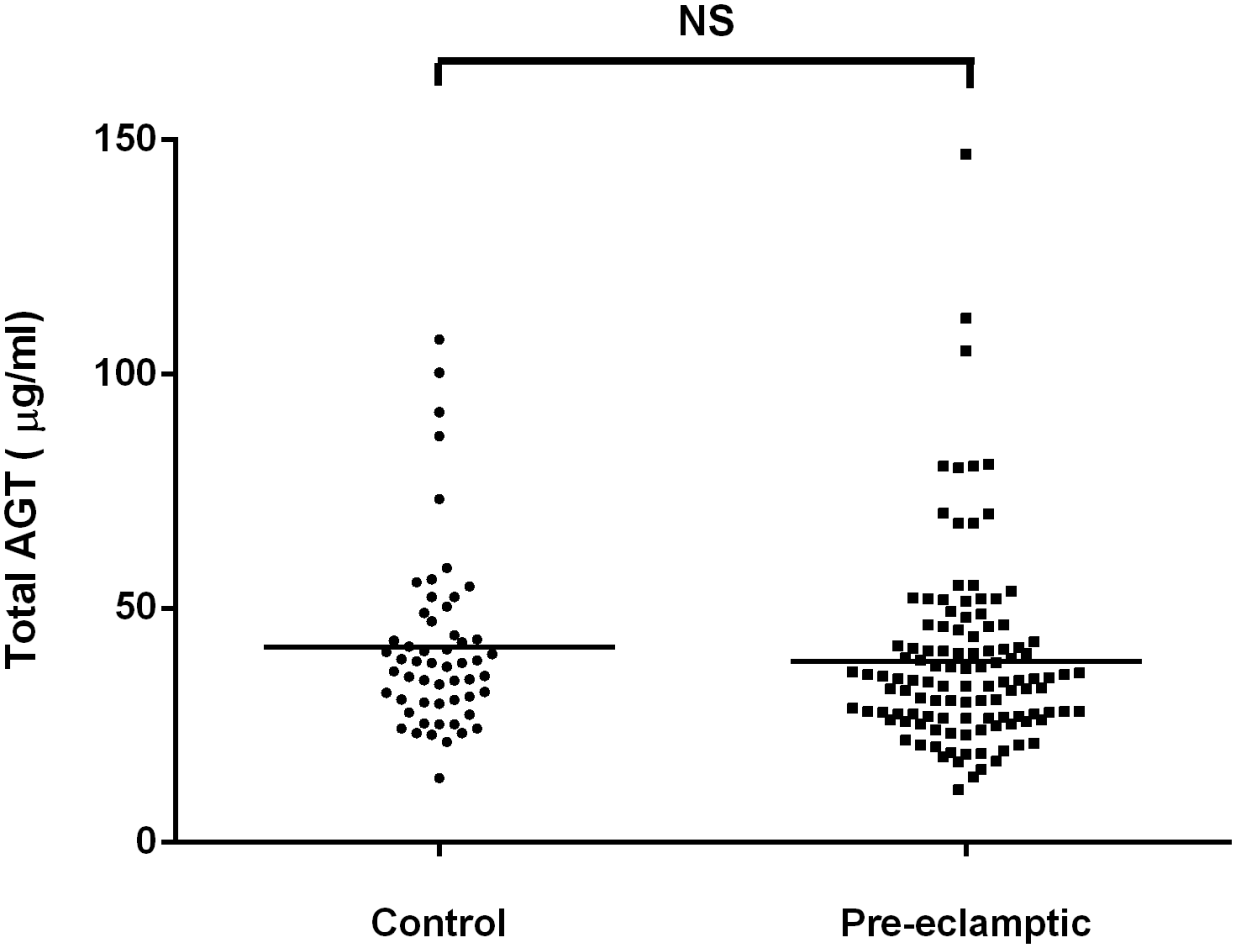
Total plasma angiotensinogen levels (μg/ml) in patients with pre-eclampsia and healthy pregnant control individuals. The amount of total plasma angiotensinogen was derived from a standard curve constructed from known concentrations of purified angiotensinogen. NS = Not Significant.

Total plasma angiotensinogen levels in the healthy pregnant control and pre-eclamptic groups did not show any statistical difference (p = 0.356) (Fig 3).

The intra-plate and inter-plate CVs for this ELISA were 3.29 ± 3.22% (mean ± SD) and 8.09%, respectively.

### C) Significantly decreased levels of free thiol angiotensinogen were demonstrated in the plasma of patients with pre-eclampsia compared to healthy pregnant controls

**Fig 4:**
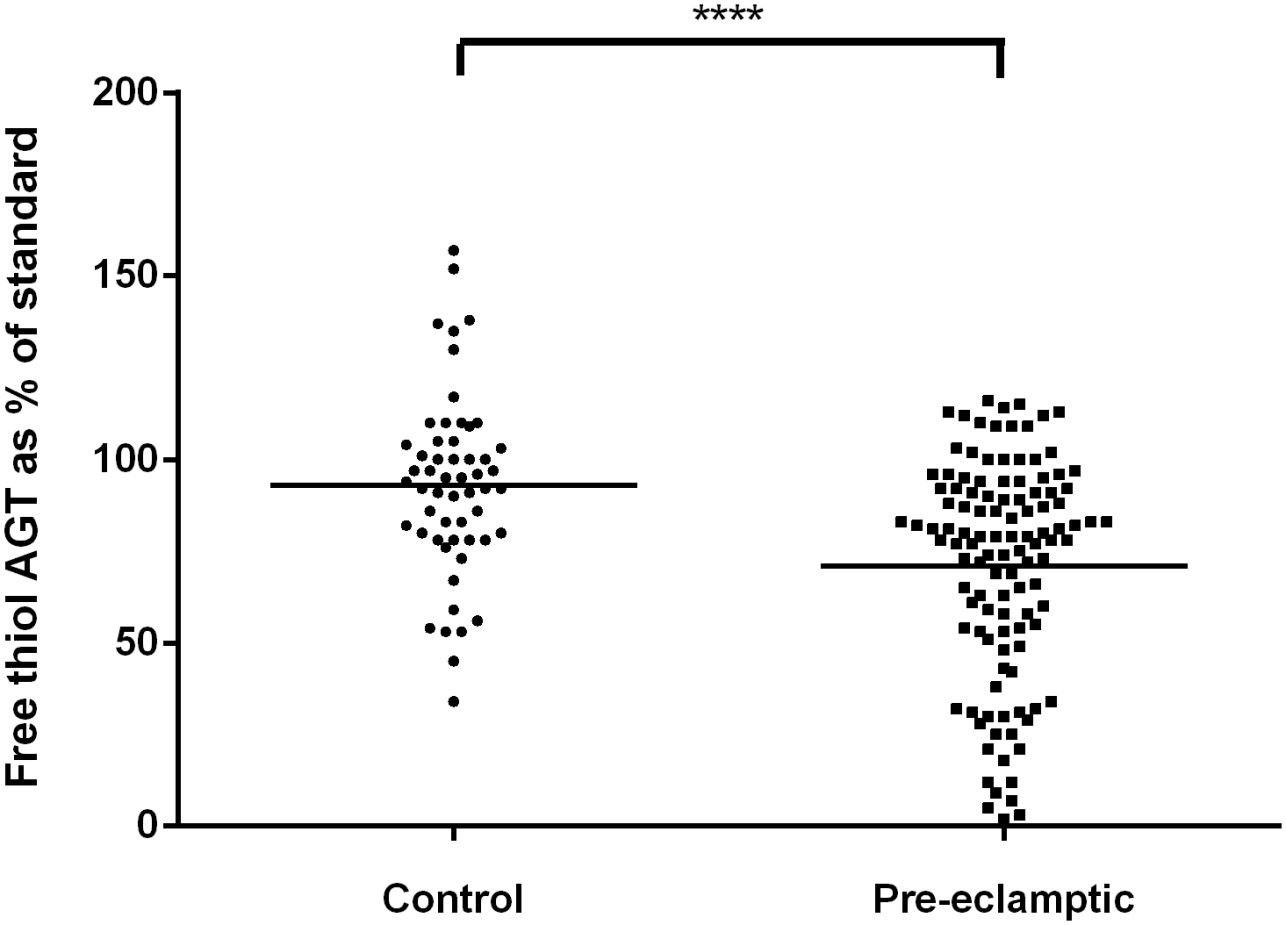
Free thiol angiotensinogen levels in patients with pre-eclampsia and healthy pregnant control individuals. Levels of free thiol angiotensinogen are expressed as a percentage of the in-house standard (**** p<0.0001).

The amount of free thiol angiotensinogen in healthy pregnant controls and patients with pre-eclampsia was expressed as a percentage of that observed in a pooled standard (derived from 420 non pregnant healthy volunteers), and compared with each other. The same in-house pooled standard was used for every MPB labeling experiment and was used in every ELISA experiment. The relative proportion of free thiol angiotensinogen, expressed as a percentage of that observed with the in-house standard, is significantly decreased in pre-eclamptic patients (70.85% ± 29.49%, n = 115) (mean ± SD) as compared to healthy pregnant controls (92.98% ± 24.93%, n = 55) (mean ± SD) *p* ≤ 0.0001 (Fig 4).

The intra-plate CV for this ELISA was 7.4 ± 6.8% (mean ± SD) and the between-plate CV was 8.7%.

### D) ROC curve analyses

**Fig 5:**
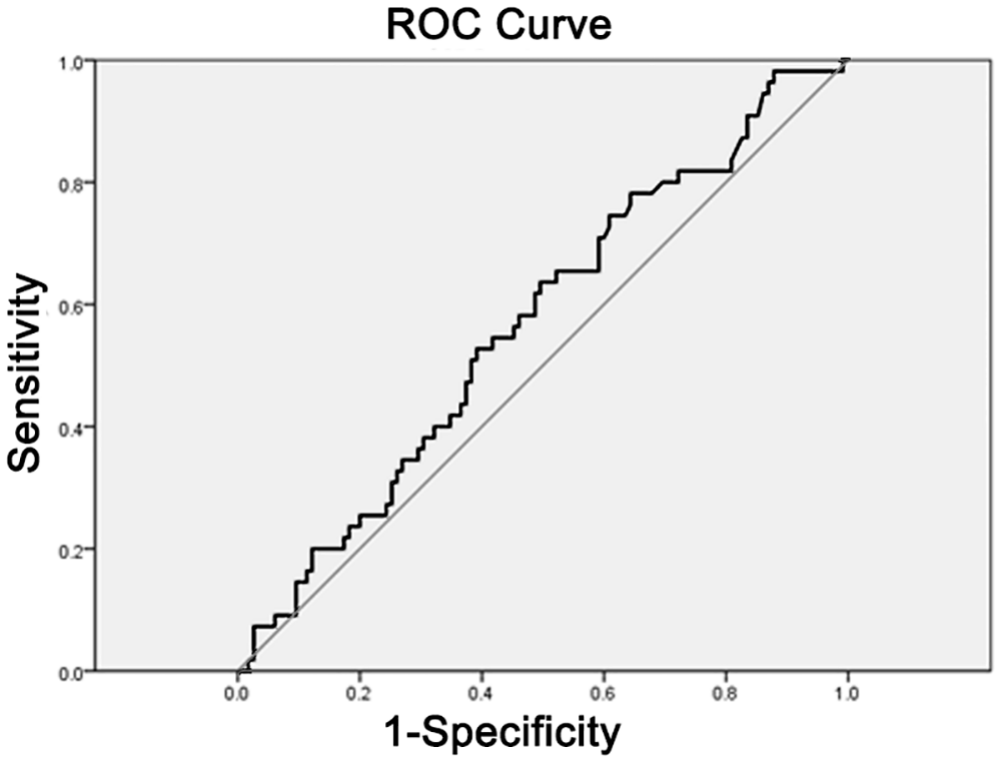
ROC curve for total angiotensinogen.

**Fig 6:**
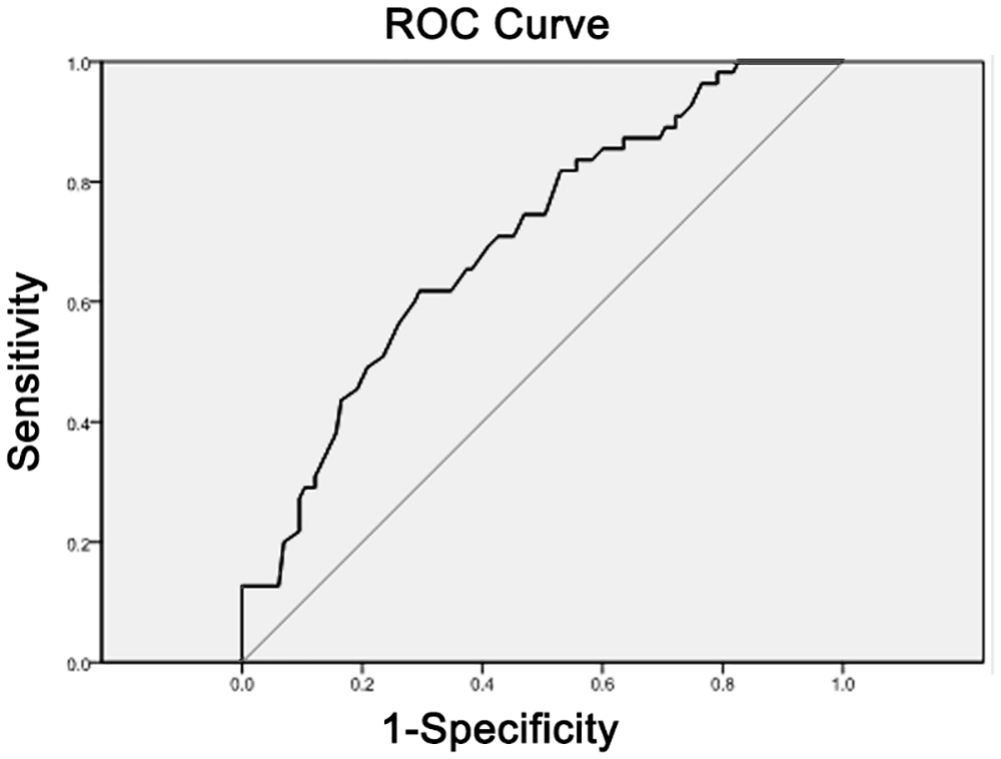
ROC curve for free thiol angiotensinogen.

The AUC, 95% confidence intervals, identified cut points, sensitivity and 1-specificity values for total and free thiol angiotensinogen (AGT) are displayed in Table 3. Figs 5 and 6 display the ROC curves for total and free thiol angiotensinogen, respectively. The free thiol angiotensinogen ROC results show that the diagnostic accuracy of the cutoff point for identifying pregnant women at risk of pre-eclampsia was higher than what would be expected by chance (AUC>0.7).

**Table 3:** ROC analysis for total and free thiol angiotensinogen.

	N	AUC	95%CI	Cutoff point	Sensitivity	1-Specificity
Total AGT	170	0.568	0.478–0.695	-	-	-
Free thiol AGT	170	0.724	0.653–0.786	75.5	0.846	0.403

## Discussion

To quantitate the level of plasma free thiol angiotensinogen, we used a highly sensitive ELISA, which entailed labeling the free thiol of angiotensinogen with a biotinylated selective probe (MPB). The MPB interacted with streptavidin attached to the wells and was detected by a specific anti-angiotensinogen monoclonal antibody (Fig 2). Our results show that the relative proportion of angiotensinogen in the free thiol form, expressed as a percentage of that observed with the in-house standard, was significantly less in pre-eclamptic patients as compared to healthy pregnant controls. The plasma levels of total angiotensinogen were not different between the two groups, which is consistent with the drop in the plasma levels of the free thiol, reduced form of angiotensinogen being due to its conversion to the oxidized form of angiotensinogen since total angiotensinogen is the sum of reduced and oxidized angiotensinogen plasma levels. Our study, with its much larger patient numbers and precise quantitative approach, significantly extends the findings of a previous small study involving only 12 pre-eclamptic individuals, whereby Zhou (10) and colleagues using Western blotting demonstrated that the ratio of the plasma levels of free thiol, reduced angiotensinogen to oxidized angiotensinogen was significantly lower in patients with pre-eclampsia compared to healthy pregnant controls [10]. Oxidized angiotensinogen may more readily predispose to hypertension associated with pre-eclampsia in view of the finding that this form releases angiotensin with greater efficiency at the cellular level than the free thiol, reduced form of angiotensinogen [10].

A pregnant woman who is aged 35 years old or greater is at an increased risk for developing preeclampsia compared to a woman aged 34 years or less [16–18]. In our study none of the participants were in this age group (mean age, 28.4±5.1 compared to 26.6±4.6 for patients and controls respectively, *p* = 0.06).

Pre-eclampsia if not treated in a timely manner can rapidly progress resulting in seizures (eclampsia), stroke, hemorrhage, kidney damage, liver failure, and death. A study by the World Health Organization has shown that hypertensive disorders of pregnancy are one of the leading causes of maternal deaths world wide [19]. We hypothesize that quantitation of the free thiol form of angiotensinogen using the ELISA method we have developed can potentially be used as a biomarker for identifying individuals at higher risk of developing pre-eclampsia, this being contingent on the undertaking of larger, multicenter, prospective studies which assess the ability of the free thiol angiotensinogen biomarker to predict pre-eclampsia weeks to months prior to the development of clinically overt disease. In this regard, the measurement of free thiol angiotensinogen plasma levels may potentially be able to be used in conjunction with other proposed biomarkers of pre-eclampsia such as soluble endoglin, soluble fms-like tyrosine kinase 1 (sflt1) and placental growth factor (PlGF) levels [20]. It is pertinent to note that a composite measure incorporating all three molecules- the ratio of (sFlt1 + soluble endoglin):PlGF is more strongly predictive of pre-eclampsia than individual biomarkers [20], and the possibility remains, pending further study, that the addition of free thiol angiotensinogen plasma levels to such a composite score may improve its predictive ability even further.

In an initial randomized controlled trial (RCT) involving women at high risk of pre-eclampsia, the anti-oxidants vitamin C and E lowered pre-eclampsia incidence by 60% [21]. Unfortunately, this effect was not confirmed in subsequent RCTs involving high [22–26] or low-risk women [26–28]. These findings suggest either that oral anti-oxidant supplementation may not be mediating its effects at the relevant tissue location, either because of inadequate penetration or dosing. It is pertinent to note that although angiotensinogen exists in a stable ratio between the free thiol and oxidized forms in the plasma pool [10], the critical interaction of renin and angiotensinogen, with respect to the control of blood pressure occurs in the renal tubules [29]. At this site the cell-surface prorenin receptor binds renin and enhances catalytic cleavage of angiotensinogen [30]. The oxidized form of angiotensinogen is preferentially bound by the prorenin receptor, thus allowing a redox-sensitive and focal modulation of angiotensin release in the kidney [10]. We hypothesize that the ELISA for free thiol angiotensinogen we have developed may allow determination of the optimal dose of anti-oxidant to be used in individuals at risk of pre-eclampsia, based on the dose that leads to normalization of levels of plasma free thiol angiotensinogen and the concomitant decrease in the plasma levels of oxidized angiotensinogen.

In summary, we have found in a large case control study that women with pre-eclampsia have lower plasma levels of the free thiol, reduced form of angiotensinogen compared to healthly, pregnant controls. Taken together with previous mechanistic functional enzymatic studies that have shown that the oxidized form of angiotensinogen may predispose to hypertension by generating angiotensin with greater efficiency compared to the reduced form of angiotensinogen [10], our study supports the notion that dysregulation of the redox status of angiotensinogen is an important contributor to the hypertension associated with pre-eclampsia, and that the measurement of the redox form of angiotensinogen may serve as a useful biomarker for predicting the development of pre-eclampsia in pregnant individuals.
